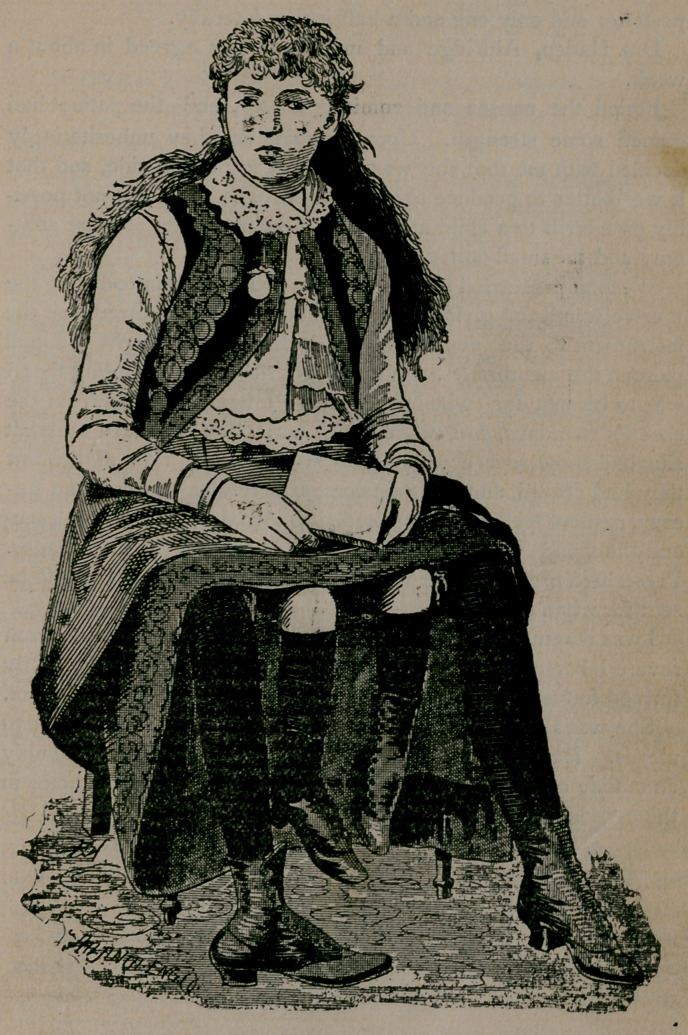# An Anomalous Case Discovered in the Female Sex

**Published:** 1888-09

**Authors:** Lewis Whaley

**Affiliations:** Blountsville, Ala.


VOL. V. SEPTEMBER, 1888. NO. 7.

### Original Communications.

### AN ANOMALOUS CASE DISCOVERED IN THE FEMALE SEX.

BY LEWIS WHALEY, M. D., BLOUNTSVILLE, ALA.

In the spring of 1887 I was called to see Mrs. B., aged 19. She had been married about one year. She gave me the history of the case as follows: Had not menstruated for two months; pain in left side, and had distressing nausea and vomiting; loss of appetite except for certain articles of diet, but everything vomited when eaten. She had headache and fever; complained of pain above pubis. She thought there was an internal abscess or tumor forming there.

Upon examination I found four inferior extremities—two sets of genital organs complete—both external and internal; two pubes, two mons veneris, two urethras, two umbilices, two distinct sets of bowels and two anni. Both genitals and bowels entirely independent of each other.

She had menstruated regularly from both sides simultaneously until two months ago. Sometimes one bowel would act when the other would not. Again one bowel would be loose, have diarrhœa, and the other be constipated.

I was uncertain as to diagnosis; therefore prescribed for symptoms and promised to see her again in a day or two. At the same time I wondered if it could be a case of pregnancy in such an anomaly. I visited her again according to promise, and found her with about the same train of symptoms, with severe paroxysms of pain in left side. Diagnosis not yet made out. I promised to call again in a few days, but before I returned I was sent for in the night. Found her with high fever; had two chills; pain still severe in left side and over left pubis; nausea and vomitiug no better. Ordered antipyrine by enema to reduce fever. Gave oxalate cerium and camph. tinct. opii to relieve vomiting and control pain, with directions to use quinine by enema when fever was down.

I found by examination that the left abdomen was becoming gradually enlarged. By digital examination and by ballotment, by comparison of the two uteri, I found the enlargment was in the left uterus. I still thought it might be pregnancy and informed her mother of my apprehensions and promised to see her again.

Returned in a few days; found she had no more chills and fever. Pain in side and bowels not so severe, but nausea and vomiting still continued.

I made another examination and gave my opinion that she was pregnant in the left side, instead of a tumor, as she had thought. She replied, "I think you are mistaken." I asked her why she thought so, and she answered, "If it had been in my right side I would come nearer believing that you are correct." I told her that I had excluded everything else and thought I was right. I also informed her that I thought it necessary that an abortion be produced, and requested consultation. Drs. Haden, of Summit, and Aldridge, of Brooksville, were sent for and an agreement made to meet in a week. Up to this time I had been visiting her at every third or fifth day for about a month. She was now becoming very much emaciated, and to prevent death from inanition and to avoid serious trouble at full term, I thought an abortion necessary, the outlet to the pelvis being two inches antero-posterior and only one and a half inches laterally.

Drs. Haden, Aldridge and myself met as agreed in about a week.

Found the nausea and vomiting better, and the patient had gained some strength. Upon examination they unhesitatingly decided with me that she was pregnant in her left side, and that it was better to produce abortion at once, as she could not possibly give birth to a child at full term, owing to her strange anatomy and the small outlet to the pelvis.

We then proceeded to bring on the abortion by introducing the uterine sound and giving fluid extract of ergot. We introduced the instrument to the depth of four inches, and thought we had pierced the membranes. We left with the understanding that I was to be sent for when necessary. I heard nothing from the case for two days, so I called to see her. She informed me that she had some severe pains soon after we left that night, but they had ceased and she was comparatively easy, except an uneasy, restless feeling. I proceeded to introduce the sound a second time, and was satisfied that I had pierced the membranes. Prescribed ergot and left with the understanding that I was to be sent for when needed.

I was summoned next day in haste. I found her suffering from severe labor-pains, and she was soon delivered of a perfectly formed fœtus of ordinary size for three and a half months' term.

She was then given tonics with instructions to use antiseptic to uterus. Her recovery was rapid and complete. This is simply an outline or synopsis of the case from memory, as the notes in the case have been misplaced.

## Figures and Tables

**Figure f1:**